# Regulation of m^6^A modification on ferroptosis and its potential significance in radiosensitization

**DOI:** 10.1038/s41420-023-01645-1

**Published:** 2023-09-15

**Authors:** Xun Chen, Lejia Zhang, Yi He, Siyuan Huang, Shangwu Chen, Wei Zhao, Dongsheng Yu

**Affiliations:** 1 Hospital of Stomatology, Guanghua School of Stomatology, Guangdong Provincial Key Laboratory of Stomatology, Sun Yat-sen University, Guangzhou, 510055 People’s Republic of China; 2 Guangdong Key Laboratory of Pharmaceutical Functional Genes, State Key Laboratory for Biocontrol, Department of Biochemistry, School of Life Sciences, Sun Yat-sen University, Guangzhou, 510275 People’s Republic of China

**Keywords:** Epigenetics, Methylation, Radiotherapy, Cell death

## Abstract

Radiotherapy is often used to treat various types of cancers, but radioresistance greatly limits the clinical efficiency. Recent studies have shown that radiotherapy can lead to ferroptotic cancer cell deaths. Ferroptosis is a new type of programmed cell death caused by excessive lipid peroxidation. The induction of ferroptosis provides a potential therapeutic strategy for radioresistance. As the most common post-transcriptional modification of mRNA, m^6^A methylation is widely involved in the regulation of various physiopathological processes by regulating RNA function. Dynamic m^6^A modification controlled by m^6^A regulatory factors also affects the susceptibility of cells to ferroptosis, thereby determining the radiosensitivity of tumor cells to radiotherapy. In this review, we summarize the mechanism and significance of radiotherapy induced ferroptosis, analyze the regulatory characteristics of m^6^A modification on ferroptosis, and discuss the possibility of radiosensitization by enhancing m^6^A-mediated ferroptosis. Clarifying the regulation of m^6^A modification on ferroptosis and its significance in the response of tumor cells to radiotherapy will help us identify novel targets to improve the efficacy of radiotherapy and reduce or overcome radioresistance.

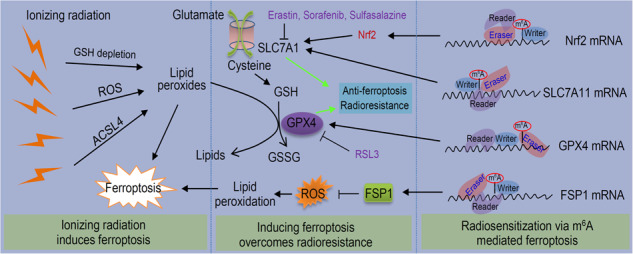

## Facts

- Radiotherapy can induce ferroptosis, a new type of programmed cell death.
- Inducting ferroptosis provides a potential therapeutic strategy for radioresistance.
- m^6^A modification is involved in the regulation of ferroptosis in cancers.
- Enhancing m^6^A-mediated ferroptosis is a promising strategy for radiosensitization.

## Open questions

- How does radiotherapy induce ferroptosis?
- How does m^6^A modification regulate ferroptosis?
- Which ferroptosis effector molecules can be regulated via m^6^A-modification to enhance ferroptosis and radiosensitivity?
- How to achieve radiosensitization through the regulation of m^6^A modification?

## Introduction

Radiotherapy is a common treatment for many kinds of cancers. However, radioresistance is a major issue, which greatly limits the clinical efficiency and prognosis of cancer patients. Overcoming radioresistance is a major challenge in cancer treatment. Therefore, it is urgent to uncover the potential mechanism leading to radioresistance and find possible solutions. Recently, several studies have shown that radiotherapy can induce ferroptosis in various types of tumors [1–4]. Ferroptosis is a type of regulated cell death triggered by unrestricted lipid peroxidation [5]. It has been found to play an important role in radiation sensitization [6–8]. Induction of ferroptosis may provide a potential therapeutic strategy for clinical radioresistance.

N^6^-methyladenine (m^6^A) modification is the most prevalent epitranscriptome modification in mammalian mRNA [9, 10]. It is widely involved in the regulation of various physiological and pathological processes by regulating RNA stability, mRNA splicing, microRNA processing and mRNA translation [11–14]. There is increasing evidence that m^6^A modification and m^6^A regulatory factors regulate the susceptibility of cells to ferroptosis, thereby affecting the radiosensitivity of tumor cells [6, 15–18]. Therefore, understanding the regulation of m^6^A modification on ferroptosis and its significance in the response of tumor cells to radiotherapy will help to find novel targets to improve the efficacy of radiotherapy and alleviate or overcome radioresistance. In this review, we will summarize the mechanism and significance of radiotherapy-induced ferroptosis, as well as the regulation of m^6^A modification on it, and discuss the radiosensitization via enhancing m^6^A-mediated ferroptosis.

## The mechanism and significance of radiotherapy-induced ferroptosis

### Ferroptosis and its regulation

Ferroptosis is a new type of iron-dependent programmed cell death triggered by lipid peroxidation, which is involved in a variety of physiopathological processes [5]. Iron is an essential trace mineral element in almost all organisms, but it can promote lipid peroxidation by catalyzing the production of reactive oxygen species (ROS) through the Fenton reaction [19, 20]. Iron can also indirectly promote ferroptosis by acting as a cofactor in the enzymes that promote lipid oxidation [5, 21, 22]. Lipid biosynthesis and metabolism are closely related to ferroptosis. The peroxidation of lipids, specifically polyunsaturated fatty acids (PUFAs), is the key driver of ferroptosis [23, 24]. Ferroptosis is induced when the peroxidation of phospholipid-PUFAs exceeds the scavenging capacity of the cell antioxidant system. The synthesis of phospholipids containing PUFAs provides substrates for peroxidation [25], which requires acyl-CoA synthetase long-chain family member 4 (ACSL4) to esterify CoA onto long PUFAs [26].

**Fig. 1:**
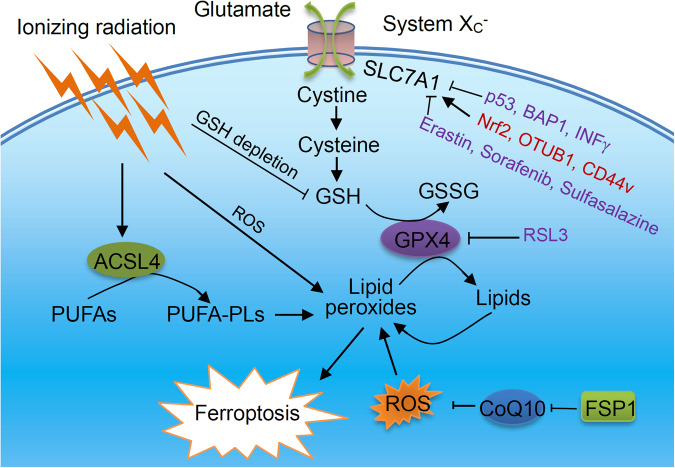
Induction of ferroptosis and radiotherapy-induced ferroptosis. Excessive production of lipid peroxides can lead to ferroptosis. GPX4 helps to eliminate lipid peroxides and exerts anti-ferroptotic effects, which requires GSH to provide reducing power. SLC7A11 imports cystine into the cell, providing the substrate for the synthesis of GSH. Ionizing radiation induces the expression of ACSL4, which provides the substrate PUFA-PLs for lipid peroxidation. Ionizing radiation-mediated ROS consumes cellular GSH and promotes lipid oxidation.

Ferroptosis is regulated by multiple factors, and solute carrier family 7 member 11 (SLC7A11) and glutathione peroxidase 4 (GPX4) are the main regulators (Fig. 1). GPX4 can use reduced glutathione (GSH) as a coenzyme to eliminate lipid peroxides, thereby inhibiting ferroptosis [27–29]. GSH depletion promotes the ferroptosis of cancer cells by reducing GPX4 activity [28, 30]. The biosynthesis of GSH requires the rate-limiting precursor cysteine (the reduced form of cystine) as the substrate. The import of extracellular cystine is regulated by cystine/glutamate antiporter (system X_C_^−^) [31], which is composed of a heavy chain (SLC3A2) and a light chain (SLC7A11) [32]. SLC7A11 is a key transporter of cysteine and introduces extracellular cystine into the cells. Cysteine is subsequently used to synthesize GSH [33].

The expression and activity of SLC7A11 can be regulated at multiple levels. SLC7A11 is a target of p53-mediated transcriptional repression [34]. Interferon-γ from CD8^+^ T cells can impair tumor cystine uptake by downregulating SLC3A2 and SLC7A11 [35]. Nuclear factor erythroid factor 2-related factor 2 (Nrf2) and kelch-like ECH-associated protein 1 (KEAP1) signalling can regulate the system X_C_^−^ and reduce ferroptosis [36]. Nrf2 is a transcription factor that promotes the transcription of SLC7A11 under oxidative stress by binding to the antioxidant response elements in its promotor region [37]. The transcription of SCL7A11 can also be regulated by the tumor suppressor BRCA1-associated protein 1 (BAP1) that encodes a nuclear deubiquitinase to reduce histone 2 A ubiquitination (H2Aub) on chromatin [38]. BAP1-mediated deubiquitination dissociates H2Aub from the SLC7A11 promoter and inhibits the expression of SLC7A11, which subsequently suppresses cystine uptake and induces ferroptosis. In addition to transcriptional regulation, the level of SLC7A11 is also regulated by posttranslational modification and stability. For example, noncanonical deubiquitinase OTU domain-containing ubiquitin aldehyde-binding protein 1 (OTUB1) interacts with SLC7A11 to prevent its degradation [39] and the adhesion molecule CD44 variant (CD44v) acts as a binding partner for stabilizing SLC7A11 [40].

Inhibition of GPX4 and SLC7A11 by corresponding inhibitors can trigger ferroptosis, so they are ferroptosis inducers (FINs). Ferroptosis suppressor protein 1 (FSP1), also known as AIFM2, is a glutathione-independent ferroptosis suppressor, which acts as a coenzyme Q10 oxidoreductase and restores the antioxidant capacity of cells [41]. Many cancer cells are sensitive to ferroptosis [27, 42, 43]. If oxidative damage caused by radiotherapy can to a certain extent lead to ferroptosis, ferroptosis inducers may be used as a radiosensitizer. Recently, ferroptosis has been recognized as an important mechanism of tumor suppression and radioresistance mediated by radiotherapy [44].

### Contribution of ferroptosis to radiotherapy

It is well known that radiotherapy can induce DNA double-strand breaks and subsequent unregulated cell death [2, 45]. Further studies found that the response of tumor to radiotherapy involves many other forms of regulated cell death, including apoptosis, necroptosis, and autophagy [46–49]. In addition to DNA damage, radiotherapy also generates ROS, which can induce oxidative damage of cell components including the lipid membrane [50, 51]. Actually, two decades ago, it was found that ionizing radiation can induce lipid peroxidation [52]. It has also been confirmed that ROS induced by radiotherapy can lead to peroxidation of PUFAs [2, 20, 53]. Excessive production of lipid peroxidation can lead to ferroptosis [5]. In recent years, the contribution of ferroptosis to radiotherapy efficacy or radiosensitization has attracted great attention [54–56]. Several studies have confirmed that ferroptosis is an important factor in the radiotherapy-induced cell death response, and ferroptosis inactivation can promote radioresistance [1–4]. Oxidative stress and ferroptosis caused by ionizing radiation are one of the most important biological effects that destroy tumors [4].

It has been proved that radiotherapy can induce ferroptosis in many cancer models, including non-small cell lung cancer (NSCLC) [1], ovarian cancer [2], fibrosarcoma, adenocarcinoma and glioma [3]. At first, it was found that ionizing radiation could induce ferroptosis in tumor cells of xenograft mice. Ferroptosis agonists enhance the efficacy of radiotherapy, while ferroptosis antagonists have the opposite effect [2]. Further research confirmed that ferroptosis is partly responsible for radiation-induced cancer cell death. Significant genetic and biochemical characteristics of ferroptosis are observed in cancer cells treated with radiation, and ferroptosis inhibitors suppress radiation-induced cell death. Radiation-induced lipid peroxidation can trigger ferroptosis in several cancer types and act in synergy with ferroptosis inducers such as system X_C_^–^ inhibitor erastin and GPX4 inhibitor RAS-selective lethal 3 (RSL3) [3]. Importantly, this effect is attributed to increased cytoplasmic lipid peroxidation, rather than the enhancement of DNA damage or caspase activation. The application of ferroptosis inducers enhances the antitumor efficacy of radiation in a murine xenograft model and in human patient-derived models [3]. The study also confirmed that radiotherapy can induce ferroptosis in cancer patients, and the increase of ferroptosis in cancer patients is related to better response to radiotherapy and longer survival periods [1]. In addition, the measurements of ferroptosis characteristic indicators, such as the expression of SLC7A11 and GPX4, as well as the intracellular lipid peroxidation and Fe^2+^ concentration, indicate that high level of ferroptosis increases the radiosensitivity of hepatocellular carcinoma [57].

The mechanism of ionizing radiation-induced lipid peroxidation and ferroptosis is not completely clear. First, excessive ROS produced by radiotherapy can promote lipid peroxidation (Fig. 1). Next, ionizing radiation induces ferroptosis partly through upregulating ACSL4 [1]. ACSL4 is a lipid metabolism enzyme required for the synthesis of phospholipids containing PUFAs. ACSL4 deficiency significantly eliminates the radiation-induced ferroptosis and leads to radioresistance. Finally, ionizing radiation also depletes intracellular GSH, which impairs the anti-ferroptosis effect mediated by GPX4 and further promotes ferroptosis [3, 55]. In previous studies, the effect of ionizing radiation on the expression of SLC7A11 was not entirely consistent. As an adaptive response, ionizing radiation induces the expression of ferroptosis inhibitors SLC7A11 and GPX4. Inactivation of SLC7A11 or GPX4 with ferroptosis inducers (FINs) can make radioresistant cancer cells and xenograft tumors sensitive to ionizing radiation, indicating the potential significance of radiotherapy combined with FINs in cancer treatment [1]. However, some studies have reported the inhibitory effect of ionizing radiation on the expression of SLC7A11 [2]. DNA damage-induced ataxia telangiectasia mutated (ATM) kinase activated by radiotherapy transcriptionally inhibits the expression of SLC7A11 to promote tumor ferroptosis. In addition, immune cells, especially CD8^+^ T cells, may participate in the induction of ferroptosis during radiotherapy. Immunotherapy can make tumors sensitive to radiotherapy by promoting ferroptosis of tumor cells. IFNγ produced by immunotherapy-activated CD8^+^ T cells can promote tumor ferroptosis and induce radiosensitization. Radiotherapy-activated ATM and IFNγ-induced STAT1 signalling jointly repress SLC7A11 to reduce cystine uptake and enhance tumor lipid peroxidation and ferroptosis [2]. This explains why radiotherapy and immunotherapy can synergistically induce tumor ferroptosis. In addition, other mechanisms mediated by DNA damage, such as mitochondrial DNA stress-activated autophagy, may contribute to ferroptosis in radiotherapy [58].

## The regulation of m^6^A modification on ferroptosis of cancers

### m^6^A modification and its regulators

**Fig. 2:**
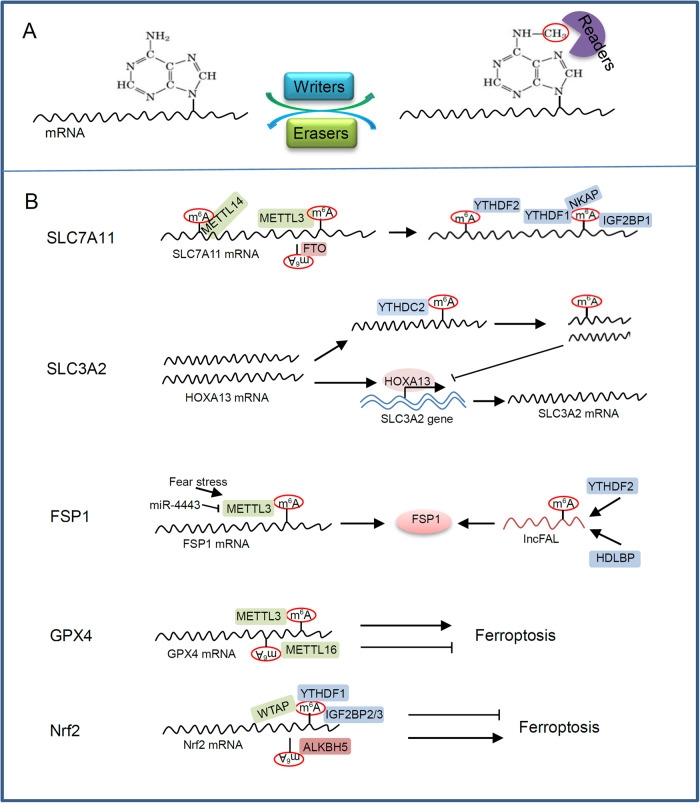
m^6^A modification and its regulation on ferroptosis of cancers. **A** The m^6^A writers and erasers cooperate to maintain a dynamic balance of m^6^A modification, while the m^6^A readers recognize m^6^A modification on RNAs and mediate downstream biological functions. **B** Several major regulatory factors of ferroptosis regulate ferroptotic cancer cell death through m^6^A modification.

N^6^-methyladenine (m^6^A) is the most common post-transcriptional modification of eukaryotic RNAs [9, 10]. Modification is a dynamic reversible process coordinated by m^6^A methyltransferases and demethylases (Fig. 2A). m^6^A methyltransferases, also known as m^6^A writers, acts in the form of complex and transfer methyl onto the nitrogen atom on amino group at the 6th position of adenine. Known m^6^A writers include methyltransferase-like (METTL) -3/14/16, Wilms tumor 1-associated protein (WTAP), RNA binding motif protein 15 (RBM15), RBM15B, vir-like m^6^A methyltransferase associated (VIRMA), and zinc finger CCCH-type containing 13 (ZC3H13). m^6^A demethylases, such as fat mass and obesity-associated protein (FTO), AlkB homolog 5 (ALKBH5) and ALKBH3, act as m^6^A erasers to remove m^6^A from modified RNAs. The m^6^A writers and erasers cooperate to maintain the dynamic balance of m^6^A modification. The m^6^A modification of RNAs can be recognized by the m^6^A reader proteins to exert different biological functions. Many proteins function as m^6^A readers, including YT521-B homology (YTH) domain-containing proteins (YTHDC) 1/2, YTH domain-containing families (YTHDF) 1/2/3, insulin-like growth factor 2 mRNA binding proteins (IGF2BP) 1/2/3, heterogeneous nuclear ribonucleoprotein (HNRNP) A2B1 and C, and eukaryotic initiation factor 3 (eIF3).

RNA m^6^A modification is involved in the regulation of RNA splicing, translation, stability, translocation, and advanced structure, which has broad biological significance [11, 12]. m^6^A modification and its regulatory genes affect many aspects of tumors [11, 12, 59]. For example, m^6^A writer METTL3-mediated signalling can promote the development and progression of tumors [60–65], and resistance [18] of tumors to chemotherapy and radiotherapy. The m^6^A demethylation of different signal molecules mediated by demethylase FTO promotes the occurrence, growth, metastasis and progression of tumors [66–72]. The biological significance of m^6^A modification is very complicated, largely depending on the targets of the modification [73, 74].

### m^6^A modification is involved in the regulation of ferroptosis

Many studies have linked m^6^A modification with programmed cell death including ferroptosis [75–78]. The system X_C_^−^ plays an important role in the control of ferroptosis. The regulation of system X_C_^−^ and its components through m^6^A modification will greatly affect cell’s ferroptosis activity. SLC3A2 and SLC7A11 are two subunits of system X_C_^−^, and are the targets of the m^6^A reader YTHDC2 to execute ferroptosis in lung adenocarcinoma cells [79, 80]. YTHDC2 can disrupt the stability of Homeo box A13 (HOXA13) mRNA in m^6^A-dependent manner. The latter can regulate the transcription of SLC3A2 subunit of system X_C_^−^ (Fig. 2B). Therefore, YTHDC2 inhibits SLC3A2 and induces ferroptosis by inhibiting HOXA13 in an m^6^A-indirect manner [80].

SLC7A11, the catalytic subunit of system X_C_^−^, is the key regulatory target of m^6^A modification. YTHDC2 destabilizes SLC7A11 mRNA in an m^6^A-dependent manner [79]. METTL3 can mediate m^6^A modification of SLC7A11 mRNA (Fig. 2B), which stabilizes SLC7A11 mRNA and promotes its translation, thus enhancing ferroptosis resistance of lung adenocarcinoma [81] and hepatoblastoma [82]. This process may need YTHDF1 [81] or IGF2BP1 [82] as the m^6^A readers of SLC7A11. IGF2BP1 can enhance SLC7A11 mRNA stability by inhibiting SLC7A11 mRNA deadenylation in an m^6^A-dependent manner [82]. METTL14 induces m^6^A modification of SLC7A11 mRNA at 5’-UTR and subsequent YTHDF2-dependent degradation. Hypoxia blocks ferroptosis of hepatocellular carcinoma by inhibiting METTL14 in a HIF-1α-dependent manner [83]. SLC7A11 has been identified as a potential FTO regulatory gene. FTO regulates ferroptosis of papillary thyroid carcinoma cells by mediating m^6^A demethylation of SLC7A11, thus preventing the progression of thyroid cancer [84]. NF-κB activating protein (NKAP) is an RNA-binding protein that acts as an inhibitor of ferroptosis. NKAP protects glioblastoma cells from ferroptosis by binding to m^6^A and promoting SLC7A11 mRNA splicing and maturation [85]. m^6^A-hypomethylation mediates upregulation of fibroblast growth factor receptor 4 (FGFR4) in anti-HER2 resistant breast cancer. FGFR4 inhibition triggers ferroptosis via the β-catenin/TCF4-SLC7A11/FPN1 axis [86].

FSP1 is another important regulatory target of m^6^A modification. miR-4443 can regulate m^6^A modification and expression of FSP1 by targeting METTL3, and subsequent FSP1-mediated ferroptosis [87]. Therefore, exosomal miR-4443 promotes cisplatin resistance in NSCLC. High-density lipoprotein-binding protein (HDLBP) binds to and stabilizes ferroptosis-associated lncRNA (lncFAL), which mediates an FSP1-dependent anti-ferroptosis in hepatocellular carcinoma (HCC) [88]. lncFAL interacts with FSP1, inhibiting Trim69-dependent FSP1 polyubiquitination degradation. YTHDF2 could promote lncFAL expression in an m^6^A-dependent manner. These results support the great potential of targeting FSP1 as a promising therapeutic approach for cancer patients [88]. Moreover, the upregulation of METTL3 induced by fear stress stabilizes FSP1 mRNA through m^6^A modification, which leads to glioma progression by inhibition of ferroptosis. The data provide a new understanding of the psychological impact on tumor development [89]. Similarly, GPX4 is also regulated by m^6^A modification. Neutrophil extracellular traps (NETs) mediate m^6^A modification and regulate sepsis-associated acute lung injury by activating ferroptosis in alveolar epithelial cells. The upregulation of ferroptosis depends on the m^6^A modification of GPX4 induced by METTL3 [90]. METTL16 enhances m^6^A modification-mediated GPX4 expression and anti-ferroptosis effect to promote breast cancer progression [91]. IGF2BP3 is highly expressed in lung adenocarcinoma and desensitizes ferroptosis in a manner that depends on its binding capacity to m^6^A-methylated mRNAs encoding anti-ferroptotic factors, including GPX4, SLC3A2, acyl-CoA synthetase long-chain family member 3 (ACSL3), and ferritin heavy chain 1 (FTH1) [92].

Another target modified by m^6^A methylation is Nrf2. WTAP can promote m^6^A modification on 3’-UTR of endogenous antioxidant factor Nrf2 mRNA and its stability by binding with m^6^A reader YTHDF1 on the m^6^A site of Nrf2 mRNA [93]. Thus, WTAP accelerates bladder cancer progression by targeting Nrf2 through m^6^A-dependent ferroptosis regulation. It was reported that RNA demethylase ALKBH5 can affect the progression of various tumors. ALKBH5 promotes ferroptosis in hypopharyngeal squamous cell carcinoma by inhibiting the expression of Nrf2 in an m^6^A-IGF2BP2-dependent manner. ALKBH5 demethylates the 3’-UTR m^6^A sites of Nrf2 mRNA. m^6^A modification and the m^6^A reader IGF2BP2 are necessary to stabilize Nrf2 mRNA [94]. ALKBH5 also suppresses the progression of thyroid cancer by reducing the m^6^A level of TIAM1 and inducing ferroptosis through m^6^A-TIAM1-Nrf2/HO-1 axis [13]. Since IGF2BP3 recognizes m^6^A modification of Nrf2 mRNA and stabilizes it, IGF2BP3 knockdown significantly promotes ferroptosis of hepatocellular carcinoma cells after administration of sorafenib [95].

In addition to directly mediating m^6^A modification of anti-ferroptotic factors such as SLC7A11 [81, 82], FSP1 [87] and GPX4, m^6^A regulatory factors also regulate ferroptosis through other different mechanisms. High glucose and high fat (HGHF)-induced ferroptosis in osteoblasts may be the main cause of osteoporosis in diabetes. Osteoblast ferroptosis is activated through the METTL3/ASK1-p38 signalling pathway to promote HGHF-induced diabetic bone loss [96]. METTL14 promotes doxorubicin (DOX)-induced ferroptosis in cardiomyocytes through regulating the KCNQ1OT1-miR-7-5p-TFRC axis. DOX induces the upregulation of METTL14, which catalyzes the m^6^A modification of the long non-coding RNA KCNQ1OT1. KCNQ1OT1, as a miR-7-5p sponge, can prevent miR-7-5p-mediated degradation of transferrin receptor (TFRC) and subsequent ferroptosis [97]. The m^6^A writer WTAP-mediated m^6^A modification on circCMTM3 inhibits hepatocellular carcinoma ferroptosis by recruiting IGF2BP1 to increase the stability of Parkinson’s protein 7 (PARK7) [98]. PARK7 shows antioxidant activity and anti-ferroptosis effect [99].

m^6^A modification may regulate ferroptosis through autophagy signaling pathway [100, 101]. The ferroptosis of hepatic stellate cells (HSCs) induced by m^6^A modification can be attributed to autophagy activation by stabilizing BECN1 mRNA via m^6^A reader protein YTHDF1 [100]. BECN1 is a key regulator of autophagy, and promotes ferroptosis through the regulation of system X_C_^−^ activity in cancer cells [102]. Dihydroartemisinin (DHA) increases the autophagy level of HSCs, thus preventing the activation of HSCs via ferroptosis pathway. The up-regulated m^6^A modification by reducing FTO is required for DHA to activate autophagy and alleviate liver fibrosis by inducing ferroptosis in HSCs [103].

On the contrary, the induction of ferroptosis will lead to changes in m^6^A modification level and m^6^A regulator activity. Our recent research has found that oxidative stress induced by the lack of an important antioxidant gene GPX8 causes reprogramming of the m^6^A epitranscriptome in oral cancer cells [104]. The m^6^A level of HSCs treated with ferroptosis inducers is enhanced by up-regulating the methyltransferase METTL4 and down-regulating the demethylase FTO [100]. Erastin can induce HSCs ferroptosis, thereby alleviating liver fibrosis in mice, while HSCs-specific inhibition of m^6^A modification can weaken erastin-induced HSC ferroptosis in murine liver fibrosis. The ferroptosis inducers may be used to prevent liver fibrosis. These studies link m^6^A with ferroptosis; therefore, targeting m^6^A to induce ferroptosis may be a promising strategy for ferroptosis-based therapy.

## Improving radiosensitivity via m^6^A-mediated ferroptosis

### Radiosensitization through inducing ferroptosis

The main mechanism leading to radioresistance of tumor cells is hypoxia. Hypoxia can also trigger ferroptosis by inducing ROS production and activate hypoxia-inducible factors [55]. Therefore, FINs-mediated ferroptosis of tumor cells may overcome the radioresistance induced by hypoxia [6]. By inducing ferroptosis, tumor cells can be re-sensitive to radiotherapy [6–8]. It was found that a ferroptosis-related gene prognostic index may predict biochemical recurrence and radiation resistance of prostate cancer patients receiving radical radiotherapy [105]. Changing the lipid composition of cell membrane by regulating lipid metabolism will affect the sensitivity of tumor cells to radiotherapy. For example, the lack of ACSL4 reduces the efficacy of radiotherapy by inhibiting the synthesis of PUFAs [1].

**Fig. 3:**
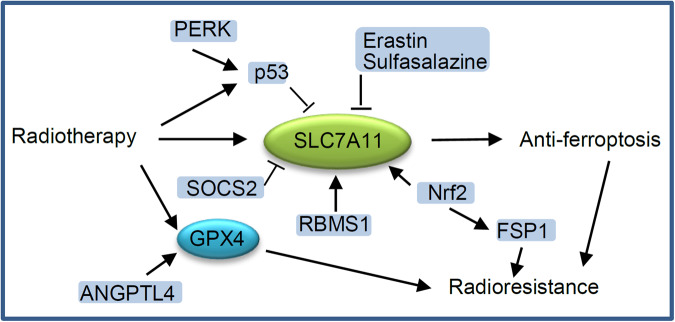
SLC7A11 and GPX4 are two key target molecules to re-sensitize tumor cells through inducing ferroptosis. The activity of SLC7A11 can be regulated by many regulatory proteins or small molecules.

SLC7A11 is a main inhibitor of ferroptosis and plays a key regulatory role in radioresistance. As mentioned above, radiotherapy can induce ferroptosis of cancer cells [55]. Radiotherapy can also inhibit ferroptosis to induce radiotherapy resistance by inducing the expression of SLC7A11 and GPX4 as a negative feedback regulatory pathway (Fig. 3). The combination of ferroptosis inducer targeting SLC7A11 and radiotherapy synergistically induces ferroptosis and improves the sensitivity of cancer cells to radiotherapy [1, 3]. Several pharmacologic inhibitors of SLC7A11, such as erastin and sulfasalazine, can increase the sensitivity of cancers to radiotherapy or re-sensitize radioresistant cancer cells [1, 3, 106]. Application of ferroptosis inducers can improve the curative effect of radiotherapy. Many proteins regulate ferroptosis and radioresistance through controlling cellular level of SLC7A11. It was reported that RNA-binding motif, single-stranded interacting protein 1 (RBMS1), an RNA-binding protein, directly binds to the translation initiation factor eIF3d to bridge the 3’- and 5’-UTR of SLC7A11, which in turn promotes the translation of SLC7A11 [107]. RBMS1 ablation promotes ferroptosis through inhibiting SLC7A11 translation and SLC7A11-mediated cystine uptake. RBMS1 depletion or inhibition of RBMS1 expression by nortriptyline hydrochloride sensitizes radioresistant lung cancer cells to radiotherapy through promoting ferroptosis [107]. The suppressor of cytokine signaling 2 (SOCS2) promotes ferroptosis and radiosensitization in cancer by enhancing the ubiquitination of SLC7A11 [57]. The expression of SOCS2 is negatively correlated with radiosensitivity of HCC and positively related to ferroptosis. In terms of mechanism, the SH2-domain of SOCS2 can specifically interact with the N-terminal domain of SLC7A11. SOCS2 acts as a bridge to transfer the attached ubiquitin to SLC7A11, and promotes K48-related polyubiquitination degradation of SLC7A11. Therefore, SOCS2 can enhance the ubiquitination degradation of SLC7A11 and promote ferroptosis, which suggests that targeting SOCS2 may improve the efficiency of radiotherapy [57]. In addition, immunotherapy can enhance the efficacy of radiotherapy, which cooperatively inhibits SLC7A11 to induce ferroptosis of tumor cells [2, 35].

SLC7A11 is also regulated by p53. p53 is the most common mutation gene in human cancers, and is also the main effector to radiotherapy. Studies have found that ferroptosis is related to p53-mediated radiosensitization. Radiotherapy-mediated p53 activation promotes irradiation-induced ferroptosis partly through antagonizing irradiation-induced SLC7A11 expression and inhibiting glutathione synthesis [44]. p53 deficiency promotes radioresistance in tumors partly through SLC7A11-mediated ferroptosis inhibition. Ferroptosis inducers that inhibit SLC7A11 can cause radiosensitization of p53-deficient tumor cells, tumor organoids and tumors. Therefore, Ferroptosis inducers combined with radiotherapy can be used to treat p53-mutant cancers [44]. In addition, PKR-like ER kinase (PERK), a sensor of unfolded protein response, facilitates ferroptosis via regulating p53 expression to down-regulate SLC7A11, contributing to the sensitivity of HCC cells to high linear energy transfer carbon ions radiation [108].

Nrf2 induces transcription of antioxidant genes, which plays an important role in resisting oxidative damage [19]. Many genes involved in cellular iron homeostasis are regulated by Nrf2. Since ferroptosis is triggered by unrestricted lipid peroxidation and iron accumulation, Nrf2 inhibition significantly increases sensitivity to ferroptosis [19]. Many Nrf2 target genes are involved in the regulation of ferroptosis [109]. At first, SLC7A11 is one of the downstream target genes of Nrf2. SLC7A11-mediated ferroptosis inhibition contributes to radioresistance. Nrf2 can directly bind to the SLC7A11 promoter region and induce the expression of SLC7A11, thereby promoting radioresistance by inhibiting ferroptosis [110]. Esophageal squamous cell carcinoma patients with high Nrf2 nuclear expression and SLC7A11 expression have poor prognosis and treatment responses [110]. FSP1 has also been identified as a transcriptional target of Nrf2, and acts as the key effector in Nrf2-mediated ferroptosis resistance and radioresistance in KEAP1 deficient lung cancer cells [109, 111]. KEAP1 is frequently mutated or inactivated in lung cancers, while KEAP1 mutant lung cancers are resistant to most therapies including radiotherapy. It is ubiquinone (CoQ)-FSP1 axis that mediates ferroptosis resistance and radioresistance in KEAP1 deficient lung cancer cells. Ferroptosis induced by pharmacological inhibition of the CoQ-FSP1 axis makes KEAP1 deficient lung cancer cells or patient-derived xenograft tumors sensitive to radiation [109, 111]. Inhibition of the Nrf2-antioxidant response element (ARE) pathway can improve the sensitivity of artesunate and eliminate the head and neck cancer resistance to ferroptosis [112]. AGuIX nanoparticles based on gadolinium have been proven to improve the radiosensitivity of cancers, which may regulate the anti-ferroptosis system by inhibiting the Nrf2-GSH-GPX4 signaling pathway [113].

Regulating GPX4 activity also affects ferroptosis and radiosensitivity of cancers. The inhibition of GPX4-mediated ferroptosis and the reduction of lipid peroxidation have been proven to be related to hypoxia-induced radioresistance. Hypoxic NSCLC cells express higher level of angiopoietin-like 4 (ANGPTL4) compared to normoxic cells. The expression level of ANGPTL4 is positively correlated with the radioresistance of NSCLC cells and xenograft tumors [114]. ANGPTL4 derived from the exosomes of hypoxic cells is ingested by adjacent normoxic cells, leading to the radioresistance of these neighbouring cells in a GPX4-dependent manner [114]. Both intracellular and exosomal ANGPTL4 contribute to hypoxia-induced radioresistance of lung cancer. Erastin decreases the radioresistance of NSCLC cells partially by inducing GPX4-mediated ferroptosis [106]. Hyperbaric oxygen can significantly enhance the ferroptosis of oral squamous cell carcinoma (OSCC) cells induced by X-ray, and re-sensitize radioresistant OSCC cells through GPX4/ferroptosis regulation [115].

In addition, the metabolism or related mechanism of iron, lipid, and amino acids also mediate the radioresistance by regulating ferroptosis. Iron metabolism is associated with ferroptosis and radiotherapy efficacy. Iron-saturated holo-Lactoferrin could increase total iron content, which induces ferroptosis in triple-negative breast cancer cells and sensitizes tumor cells to radiotherapy [116]. Stearoyl-CoA desaturase (SCD1) is an enzyme responsible for the formation of oleic acid and palmitoleic acid. It was reported that targeting SCD1 can enhance radiation-induced ferroptosis and immunogenic cell death, thus improving radiation sensitivity [117]. SCD1 inhibitors can induce ferroptosis by reducing the formation of monounsaturated fatty acids. Inhibition of SCD1 makes tumor cells sensitive to radiotherapy and inhibits the growth of esophageal squamous cell carcinoma in vivo. The metabolomics analysis of irradiation-resistant HepG2 cells shows that the intracellular amino acids, especially N-acetylglutamine, increase significantly during the stress of ferroptosis [4]. N-acetylglutamine is a derivative of glutamine, which plays an important role in maintaining redox homeostasis. Glutamine starvation can significantly promote ferroptosis, and vice versa. Bioinformatics analysis based on TCGA data indicates that the glutamine transporter SLC1A5 is an independent prognostic amino acid-ferroptosis gene. The knockdown of SLC1A5 promotes lipid peroxidation and irradiation-mediated oxidative damage. The results indicate that SLC1A5 may be a potential target for radioresistance as an anti-ferroptosis gene [4]. The stem cell characteristics of tumor cells are also related to the resistance of radiotherapy and ferroptosis. It was found that the spheroids with the stem cell-like traits formed by nasopharyngeal carcinoma (NPC) cells exhibit a certain degree of radioresistance and ferroptosis resistance, while itraconazole partially reverses the radioresistance of NPC spheroids through inducing ferroptosis [118].

miRNAs can also affect radiation resistance by regulating ferroptosis. The expression of miR-7-5p in clinically relevant radioresistant cells is up-regulated, and the radioresistance is lost after miR-7-5p knockdown [119]. Knockdown of miR-7-5p increases ROS production and ferroptosis, characterized by increased intracellular Fe_2_^+^ amount, up-regulation of ferroptosis marker gene expression, and excessive production of lipid peroxides [120]. These indicate that miR-7-5p controls radioresistance by producing ROS, which can lead to ferroptosis.

### Radiosensitization through m^6^A regulation

Concurrent radiotherapy and chemotherapy is the most common treatment after surgery [121]. Radiotherapy is an effective treatment for many kinds of cancers, and radioresistance is the main reason for local treatment failure. However, the potential mechanism and valuable markers of radioresistance have not been well established [17]. m^6^A modification plays an important role in gene expression regulation. Although m^6^A modification is involved in the development of tumor, its role in therapeutic resistance is still unclear. Understanding the effect of m^6^A modification on radiation response is of great significance for finding new targets and improving tumor treatment. Considering that m^6^A modification is involved in the regulation of ferroptosis and ferroptosis contributes to radiosensitivity, m^6^A methylation will regulate the efficacy of radiotherapy. In fact, some studies have shown that m^6^A RNA modification contributes to the regulation of radiotherapy resistance [6, 15, 16]. The dysregulated expression of many m^6^A enzymes, including demethylase FTO, methyltransferase METTL3 and WTAP, mediates the development of resistance of cancer cells to chemotherapy and radiotherapy [122, 123].

**Fig. 4:**
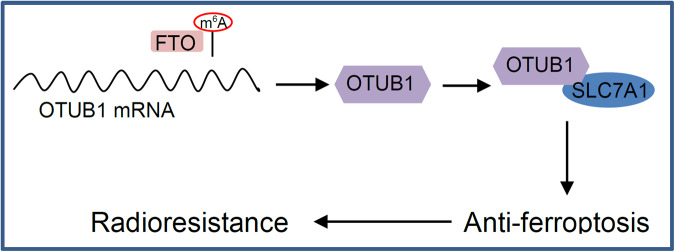
The m^6^A regulatory factor promotes tumor resistance to radiotherapy by suppressing radiation-induced ferroptosis. The demethylation of OTUB1 transcript by FTO promotes the expression of OTUB1, which inhibits ferroptosis through binding to SLC7A11 and stabilizing it.

It was reported that m^6^A demethylase FTO enhances the radioresistance of NPC via promoting deubiquitylase OTUB1-mediated anti-ferroptosis [17]. The OTUB1 can mediate ferroptosis via the stabilization of SLC7A11 in human cancer [39]. The inhibition of OTUB1 on ferroptosis depends on the interaction between OTUB1 and SLC7A11 [17]. FTO, as an m^6^A demethylase, erases the m^6^A modification of the OTUB1 transcript, which up-regulates the expression of OTUB1 and leads to radiotherapy resistance of NPC [17]. The expression of FTO in radioresistant NPC tissues and cells is significantly higher than that of its parental radiosensitive tissues and cells. Accelerating ferroptosis by FTO inhibitor or ferroptosis inducer overcomes the radioresistance of NPC patient-derived xenografts [17]. This is the first report that m^6^A regulator can promote tumor resistance to radiotherapy by suppressing radiation-induced ferroptosis (Fig. 4), suggesting that m^6^A regulator may serve as a potential therapeutic target and prognostic biomarker. It was reported that FTO can also enhance the radiotherapy resistance of cervical squamous cell carcinoma through regulating expression of β-catenin by reducing m^6^A levels in its mRNA transcripts [124].

Methyltransferase METTL3-mediated m^6^A modification plays a critical role in the development and maintenance of radioresistance. Cell response to ultraviolet-induced DNA damage can induce RNA m^6^A modification, which is regulated by METTL3 and FTO. METTL3 knockdown impairs the repair of irradiation-induced DNA damage and improves therapeutic sensitivity, suggesting the importance of m^6^A modification in the irradiation-mediated DNA damage response [125]. The expression of METTL3 is increased in glioma stem-like cells (GSCs), which plays an important role in the maintenance and radioresistance of GSCs by regulating m^6^A modification of SOX2 mRNA [126]. METTL3-mediated m^6^A mRNA contributes to the resistance of pancreatic cancer and NSCLC to radiotherapy [18, 127]. After carbon ion radiotherapy, the level of METTL3 and its mediated m^6^A modification in NSCLC cells is increased. METTL3-mediated mRNA m^6^A modification inhibits the decay of H2A histone family member X (H2AX) mRNA and enhances its expression, thus facilitating DNA damage repair and cell survival [127].

Several studies have also proved the regulatory effect of m^6^A readers on radioresistance. For example, YTHDC2 promotes radiotherapy resistance of NPC cells by activating the IGF1R/ATK/S6 signalling axis. YTHDC2 is consistently highly expressed in radioresistant NPC cells, and its expression is associated with the therapeutic effect of radiotherapy. YTHDC2 can bind to insulin-like growth factor 1 receptor (IGF1R) mRNA and promote translation initiation of IGF1R mRNA, which in turn activates the IGF1R-AKT/S6 signalling pathway [128]. YTHDF3 accelerates the translation of the DNA repair protein RAD51 homologue 4 (RAD51D) in an m^6^A-dependent manner, thereby mediating effect of hepatocyte nuclear factor 1-alpha (HNF1α) on radioresistance of cervical cancer. HNF1α is significantly up-regulated in radioresistant cervical cancer, thus promoting the resistance of cervical cancer cells to radiation. HNF1α enhances the transcription of YTHDF3, and YTHDF3 subsequently promotes m^6^A modification of RAD51D mRNA [129]. We must note that most of the above studies have shown to a large extent that radiotherapy can be sensitized through m^6^A regulation, but only a few studies have attributed radiosensitization to ferroptosis mediated by m^6^A modification [17].

Since the radiosensitivity of tumor cells can be regulated by m^6^A modification, targeting m^6^A regulators has shown great potential in tumor therapy [130, 131]. Many studies have attempted to identify small molecule inhibitors associated with m^6^A. Some compounds have been reported as potential METTL3 inhibitors [132, 133]. The anti-HIV drug elvitegravir has also been identified as a novel inhibitor of METTL3, which can inhibit the metastasis of esophageal carcinoma [134]. STM2457 is the first METTL3 catalytic inhibitor with high affinity for METTL3 [135]. It can reduce overall m^6^A levels and mRNA translation efficiency, and inhibit the growth of acute myeloid leukemia (AML). STM2457 exhibits a synergistic effect with PD-1 antibody in colorectal cancer [136]. Some small molecules can inhibit demethylase activity of m^6^A eraser proteins. Several FTO inhibitors have been identified, such as rhein [137], N-CDPCB [138], CHTB [139], and meclofenamic acid [140] and its derivatives [141, 142]. For example, rhein can disrupt the binding of FTO to m^6^A RNAs by competitively binding to the FTO catalytic domain [137]. Meclofenamic acid specifically inhibits FTO by competitively binding to FTO sites of m^6^A-modified oncogenic mRNAs, thus effectively inhibiting cancer cell proliferation [140]. Some small molecule inhibitors of FTO can exert effective anti-tumor effects by making cancer cells sensitive to the cytotoxicity of T cells [143]. A compound with anti-ALKBH5 catalytic activity has been identified to inhibit the proliferation of leukemia cells [144]. BTYNB has been shown to block the interaction between m^6^A reader IGF2BP1 and its substrate RNAs [145], leading to cancer cell cycle arrest [146]. Cucurbitacin B and 7773 block different RNA binding domains of IGF2BP1 respectively and regulate different downstream targets [147, 148]. CWI1-2 and JX5 have been identified as IGF2BP2 inhibitors, which selectively disrupt the binding of IGF2BP2 to its m^6^A modified target RNAs and exhibit good anti-leukemia activity [149, 150].

Before these m^6^A related inhibitors enter clinical application, further comprehensive research is needed, especially to strengthen in vivo research and clinical trials. Currently, only a few studies have conducted similar work. For example, the treatment of mice with R-2-hydroxyglutarate (R-2HG), a FTO inhibitor, can inhibit FTO demethylase activity, thus increasing the overall m^6^A levels and leading to anti-tumor effects in vivo [151]. METTL3 inhibitor STM2457 impairs engraftment and prolongs survival in AML mouse model [135]. METTL3 inhibitor STC-15 has entered the first stage of clinical trials [131]. In addition, the role or significance of these inhibitors in radiotherapy and radioresistance is not yet well understood. It is still unclear whether the role of these inhibitors is related to the ferroptosis. Due to the context-dependent epigenetic characteristics of cancers, it is also necessary to improve the target selectivity of inhibitors. However, some m^6^A related inhibitors have been shown to inhibit cancer progression, indicating that m^6^A could be a target for cancer therapy.

## Conclusions

Radiotherapy is widely used to treat cancer, but often leads to resistance. The well-known mechanisms of radiation resistance include activation of DNA repair and inhibition of cell apoptosis. Therefore, it is valuable to make cancer cells sensitive to radiation by alternative cell death pathways [3]. Recently, it has been confirmed that lipid peroxidation can trigger ferroptosis, which is also a major feature of cell death induced by radiotherapy and contributes to radiosensitivity [1–4]. The combination of ferroptosis inducers and radiotherapy will greatly improve the efficacy of radiotherapy. Importantly, many anti-tumor drugs have been found to cause ROS accumulation, oxidative stress, and subsequent ferroptosis in tumor cells. For example, cisplatin can trigger ferroptosis by directly consuming intracellular GSH and inhibiting GPX4 [152]. Temozolomide induces ferroptosis by promoting the expression of SLC7A11 [153]. Sorafenib can inhibit system X_C_^−^ or regulate HIF-1α/SLC7A11 pathway to promote ferroptosis [154, 155]. Therefore, they can be used as ferroptosis inducers. This can help us further understand the role of concurrent chemotherapy during radiotherapy and guide us in selecting appropriate chemotherapy drugs. As the most common post-transcriptional modification, m^6^A methylation regulates ferroptosis through different effectors and affects the sensitivity of tumor cells to radiotherapy. This makes various m^6^A regulatory molecules a promising therapeutic target in radiotherapy. Radiosensitization through m^6^A-mediated ferroptosis may be an alternative method for improving the efficacy of radiotherapy in the future.

## Data Availability

Correspondence and requests for data should be addressed to DY.
